# Two new species of the genus *Anillinus* Casey (Coleoptera, Carabidae, Anillini) from the southern United States

**DOI:** 10.3897/zookeys.1016.61397

**Published:** 2021-02-11

**Authors:** Igor M. Sokolov

**Affiliations:** 1 Systematic Entomology Laboratory, ARS, USDA, c/o Smithsonian P.O. Box 37012, National Museum of Natural History, Washington, DC 20013-7012, USA National Museum of Natural History Washington, DC United States of America

**Keywords:** Alabama, *Anillinus*, distribution, Louisiana, new species, soil fauna

## Abstract

Two new species of blind ground beetles are described from the southern United States. One species, *Anillinusrelictus***sp. nov.** (type locality: E of Oneonta, Blount County, Alabama), based on the structure of male genitalia, is similar to Texan *Anillinus*, in particular to the endogean *A.sinuatus* Jeannel. The second species, *A.felicianus***sp. nov.** (type locality: 4 mi SW Jackson, West Feliciana Parish, Louisiana), is superficially similar to the endogean *A.sinuaticollis* Jeannel from Roane County, Tennessee, and represents the first record of the genus for the state of Louisiana. All species are illustrated with digital images of habitus, body parts, and male and female genitalia. Biogeographical and evolutionary implications of the new findings are discussed.

## Introduction

The genus *Anillinus* Casey, in spite of its wide range, remains one of the most incompletely known genera of carabid beetles in the United States. Litter, soil-dwelling or cavernicolous representatives of the genus inhabit a huge area from the Potomac River in the north to the Florida Panhandle in the south, and from eastern Texas in the west to the Piedmont hills of North Carolina in the east (Bousquet 2012). Across this territory, the number of species recorded in neighboring states varies greatly, and the necklace of the Gulf States is exactly the region where the biodiversity of *Anillinus* changes from state to state rather unevenly and often unexpectedly. Moving from the west to the east, the number of species recorded to date starts from seven in Texas, then jumps down to zero in Louisiana and to one in Mississippi; thereafter, the number of species increases again to twelve in Alabama, and drops down to two in Georgia and Florida (Bousquet 2012; Sokolov et al. 2014; Sokolov 2020). Obviously, such an uneven distribution of anilline species across neighboring states may result from the interaction of natural ecological and historical factors. One more reason for the dramatic variation in the recorded numbers of species is insufficient sampling of representatives of the genus, which are hard to collect. Each discovery of a new species of *Anillinus* in the Gulf states adds to our knowledge of local faunas and brings us closer to understanding the roles of the aforementioned factors in shaping biodiversity on the lands bordering the Gulf of Mexico. Descriptions of two new species discovered in the Gulf states form the main content of this paper.

## Material and methods

This study is based on examination of specimens of *Anillinus* either collected in Louisiana or originating from the late Tom C. Barr’s collection of Anillini (now in the Carnegie Museum of Natural History, Pittsburgh, Pennsylvania, USA – **CMNH**). Type material of the newly described species is deposited in the National Museum of Natural History, Washington, DC (**NMNH**), in the CMNH, and in the Louisiana State Arthropod Museum (**LSAM**). Verbatim label data are given for the type specimens of the newly described species, with label breaks indicated by a backslash (“\”).

Terms used in this paper follow Sokolov et al. (2014).

Extractions and processing of genitalia were made using the standard techniques described by Sokolov and Kavanaugh (2014).

Photographs of the external features of the new species were taken with a Macropod Pro photomacrography system (Macroscopic Solutions, LLC). Digital images of genitalia were taken with a Nikon light microscope Eclipse N*i*-U supplied with DS-Fi2 camera and DS-LR3 camera control unit. Line drawings of selected body parts were prepared with the help of a camera lucida attached to an Olympus BX 50 compound microscope.

All specimens were measured using the tpsDig 2.17 (Rohlf 2013) software on digital photographs. Measurements for various body parts are coded as follows: **ABL** = apparent body length from clypeus to apex of elytra; **WH** = width of head at level of first orbital setae; **WPm** = maximal width across pronotum; **WPa** = width across anterior angles of pronotum; **WPp** = width across posterior angles of pronotum; **LP** = length of pronotum from base to apex along midline; **WE** = width of elytra at level of 2^nd^ discal setae; **LE** = length of elytra from apex of scutellum to apex of left elytron. ABL measurements are given in mm; others are presented as nine ratios: mean widths – WH/WPm and WPm/WE; and body parts – WPa/WPp, WPm/WPp, WPm/LP, WE/LE, LP/LE, LE/ABL and WE/ABL. All values are given as mean ± standard deviations.

## Taxonomic Part

### Order Coleoptera Linnaeus, 1758

**Family Carabidae Latreille, 1802**

**Subfamily Trechinae Bonelli, 1810**

#### Tribe Anillini Jeannel, 1937

##### Genus Anillinus

Taxon classificationAnimaliaColeopteraCarabidae

Casey, 1918

002A9E7D-23C6-51B8-A397-282EACAFF600

Anillinus Casey, 1918: 167. Type species: Anillus (Anillinus) carolinae Casey, 1918, by original designation.
Micranillodes Jeannel, 1963a: 57. Synonymy established by Bousquet (2012: 699) and confirmed by Sokolov et al (2014: 83). Type species: Micranillodesdepressus Jeannel, 1963a, by original designation.
Troglanillus Jeannel, 1963b: 147. Synonymy established by Barr (1995: 240). Type species: Troglanillusvalentinei Jeannel, 1963b, by original designation.

##### Anillinus felicianus sp. nov.

Taxon classificationAnimaliaColeopteraCarabidae

4F0B7AC4-707B-51B1-9461-F7B6FB837A01

http://zoobank.org/CA1608B7-D169-418F-9F80-02E88AEBA5B3

Figs 1 , 2 , 5

###### Type material.

***Holotype*** male (NMNH), dissected, labeled: \ USA-LA: West Feliciana Par., ~4mi SW Jackson at 30.794°N, 91.254°W, mixed pine-hardwood forest, soil washing/berlese Sokolov I.M. 19–25 Apr 2018 \. ***Paratype***: one female, labeled as holotype (NMNH); three males and three females labeled: \USA, LA, W. Feliciana Par. Feliciana Preserve Natural Area, Orange Trail, 30.792649°N, 91.253382°W, 29 Oct. 2015 \ Soil washing in hardwood forest B.E. Owens and C.E. Carlton \(LSAM).

###### Etymology.

The name of this species is a Latinized adjective based on the name of Feliciana Preserve, in which this species occurs. Feliciana Preserve is a privately owned nature reserve created by several professors of the Louisiana State University (principal developer Dr. Dorothy Prowell) and located in the Tunica Hills area of southeastern Louisiana.

###### Type locality.

USA, Louisiana, West Feliciana Parish, Tunica Hills, 4 mi SW of Jackson.

###### Recognition.

Adults of *A.felicianus* can be distinguished from those of other subterranean members of *Anillinus* by the combination of smooth pronotum and completely microsculptured head. Males of *A.felicianus* can be also distinguished from those of other congeners by the structure of the median lobe.

**Figure 1.**
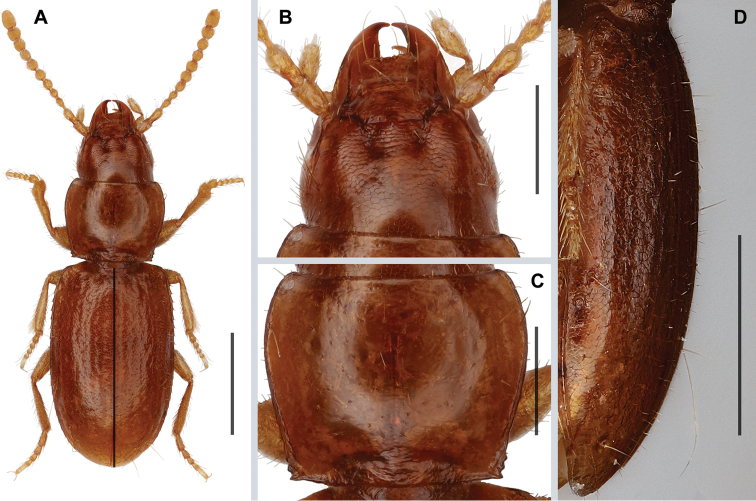
Digital images of external features of *Anillinusfelicianus* sp. nov. (male, 4 mi SW of Jackson, West Feliciana Parish, Louisiana) **A** habitus, dorsal aspect **B** head, dorsal aspect **C** pronotum, dorsal aspect **D** elytral vestiture, left lateral aspect. Scale bars: 0.5 mm (**A, D**); 0.2 mm (**B, C**).

###### Description.

Moderate-sized for genus (ABL 1.59–1.68 mm, mean 1.64±0.064 mm, n = 2).

***Habitus***: Body form (Fig. 1A) moderately convex, subparallel, elongate (WE/ABL 0.36±0.001), head moderately large in comparison to pronotum (WH/WPm 0.77±0.008), pronotum of moderate width in comparison to elytra (WPm/WE 0.82±0.006).

***Integument***: Body color brunneo-rufous, appendages testaceous. Microsculpture (Fig. 1B–D) present across all head and elytra, where it is represented by isodiametric polygonal sculpticells; and absent from disc of pronotum. Body surface shiny, surface sparsely and finely punctate, covered with sparse, yellowish, short setae. Vestiture of elytra (Fig. 1D) short (~0.3 length of discal setae). Elytral chaetotaxy typical for *Anillinus*, umbilicate series of type A (*sensu*Jeannel 1963a and Giachino and Vailati 2011): nine setae arranged in three groups, subhumeral (3+1), middle (2) and subapical (1+2), with the last two (8^th^ and 9^th^) pores of umbilicate series “geminate”, much closer to each other than 7^th^ pore is to 8^th^; in subapical group the 8^th^ pore is the longest.

***Prothorax***: Pronotum (Fig. 1C) moderately convex, of moderate size (LP/LE 0.41±0.010) and moderately transverse (WPm/LP 1.26±0.009), with lateral margins almost rectilinearly and moderately constricted posteriorly (WPm/WPp 1.26±0.009). Anterior angles indistinct, posterior angles slightly obtuse (105–110°). Width between posterior angles equals width between anterior angles (WPa/WPp 0.97±0.001). Basal margin slightly concave in middle.

***Scutellum***: Externally visible, triangular, with pointed apex.

***Elytra***: Slightly convex, of average length (LE/ABL 0.58±0.012) and width (WE/LE 0.63±0.015) for genus, with traces of 4–5 striae. Humeri distinct, rounded, in outline forming obtuse angle with longitudinal axis of body. Lateral margins subparallel in middle, slightly convergent at basal fourth, evenly rounded to apex at apical third, with shallow subapical sinuation. Basal margination distinct.

***Legs***: Protarsi of male with moderately dilated tarsomere 1. Profemora moderately swollen. Metafemora unmodified.

**Figure 2.**
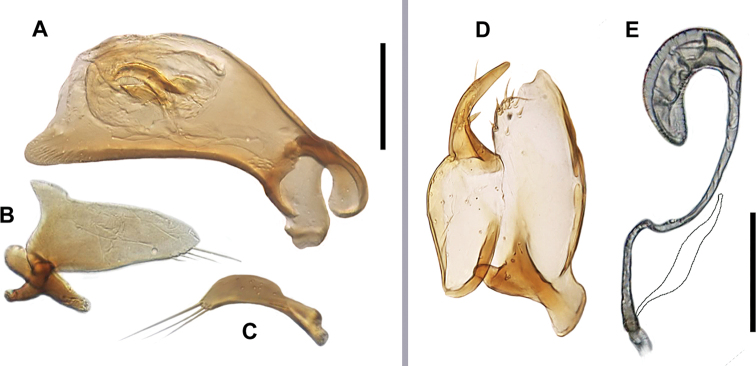
Digital images of male and female genitalia of *A.felicianus* sp. nov. (4mi SW of Jackson, West Feliciana Parish, Louisiana). Male genitalia **A** median lobe, right lateral aspect **B** left paramere, left lateral aspect **C** right paramere, right lateral aspect. Female genitalia **D** ovipositor sclerites **E** spermatheca (spermathecal gland manually restored). Scale bars: 0.1 mm.

***Male genitalia***: Median lobe (Fig. 2A) of aedeagus anopic, slightly arcuate and moderately twisted. Shaft dilated in apical two-thirds, with moderately elongate apex, slightly tapered to rounded tip. Ventral margin of median lobe straight, not enlarged, with few poriferous canals, curved downward close to basal orifice. Endophallus with dorsal copulatory sclerites fused to form slightly curved dorsal blade-like structure and straight, apically-pointed ventral plate of moderate length. Spines and scaled membranous folds of endophallus absent. Left paramere (Fig. 2B) of shape common in genus, paramere apex with four setae getting longer toward apex. Right paramere (Fig. 2C) of moderate length, bearing three long setae of approximately same length as paramere.

***Female genitalia***: Spermatheca (Fig. 2E) unsclerotized, shaped as a question mark, with sharply dilated bean-like distal part. Length of spermathecal gland shorter than length of spermatheca. Spermathecal duct uncoiled. Gonocoxites and laterotergite as in Fig. 2D. Gonocoxite 2 falciform, more than 2 times longer than wide basally, with acute ensiferous setae. Laterotergite with 7–8 setae.

###### Geographic distribution.

This species is known only from the type locality in the Tunica Hills area of West Feliciana Parish, Louisiana (Fig. 5, green circle).

###### Habitat.

All specimens of this species were collected from loess soil samples using soil washing techniques (Southwood and Henderson 2000). These samples were taken under forest canopy on the top of a hill between two gullies with temporal creeks. *Anillinusfelicianus* is a true endogean species and has never been found in litter samples.

###### Relationships.

The species belongs to group V of endogean species (*sensu*Sokolov et al. 2004), characterized by a combination of a completely microsculptured head and smooth disc of pronotum. Within this group, *A.felicianus* is most closely related to the endogean *A.sinuaticollis* Jeannel (Jeannel 1963b), differing from the latter by a smooth – not microsculptured – base of the pronotum, a bigger size, and details of the pronotum shape and body proportions. The range of *A.sinuaticollis* is confined to Roane County of Tennessee (Fig. 5, green area with vertical line pattern); thus, its range lies about 500 miles north of the type locality of *A.felicianus*.

##### Anillinus relictus sp. nov.

Taxon classificationAnimaliaColeopteraCarabidae

588729DA-5AFB-535A-9D11-D17A06E306A6

http://zoobank.org/8F185D84-D2A4-464B-97F9-A5FEDD791B47

Figs 3 , 4A–C , 5

###### Type material.

***Holotype***, one male (CMNH), dissected, labeled: \ ALABAMA: Blount Co., Tidwell Hollow Nature Trail east of Oneonta. T. N. King April 1 1972 \ 4/1/72 o [handwritten] \ THOMAS C. BARR COLLECTION 2011 Acc. No. 38,014 \. ***Paratype***, one female, labeled as holotype (CMNH).

###### Etymology.

The specific epithet is a Latin adjective, *relictus* (from Latin: abandoned, forsaken), in the masculine form, and refers to the geographical isolation of this species from its morphologically closest congeners, as it is believed to be the only remaining eastern representative of an ancestral group once more widespread.

###### Type locality.

USA, Alabama, Blount County, the Oneonta area.

###### Recognition.

Adults of A.relictus can be distinguished from those of other members of eastern *Anillinus* by the combination of the large size, completely microsculptured head and pronotum, and, especially, by the long elytral vestiture equals to 0.5–0.7 of length of discal elytral setae.

###### Description.

Large-sized for genus (ABL 2.29–2.42 mm, mean 2.36±0.092 mm, n = 2).

**Figure 3.**
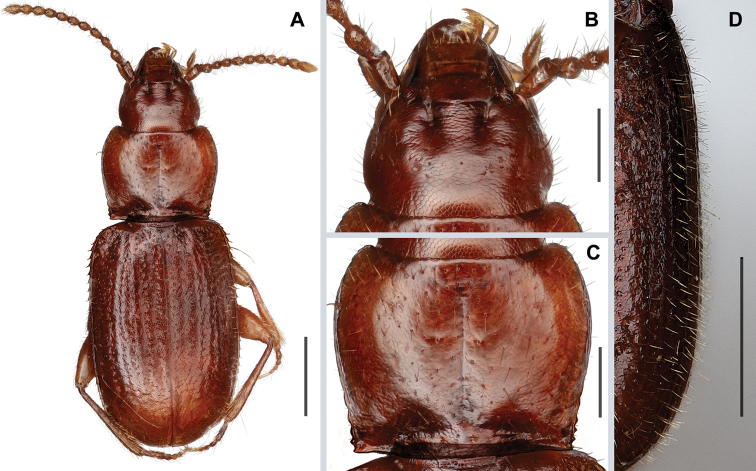
Digital images of external features of *Anillinusrelictus* sp. nov. (male, E of Oneonta, Blount County, Alabama) **A** habitus, dorsal aspect **B** head, dorsal aspect **C** pronotum, dorsal aspect **D** elytral vestiture, left lateral aspect. Scale bars: 0.5 mm (**A, D**); 0.2 mm (**B, C**).

***Habitus***: Body form (Fig. 3A) moderately convex, ovoid (WE/ABL 0.39±0.012), head of average proportions for genus (WH/WPm 0.71±0.023), pronotum moderately narrow in comparison to elytra (WPm/WE 0.77±0.007).

***Integument***: Body color piceo-brunneus, appendages testaceous. Microsculpture (Fig. 3B–D) present across all head, pronotum, and elytra, where it is represented by isodiametric polygonal sculpticells. Body surface shiny, surface sparsely and finely punctate, covered with moderately dense, yellowish, long setae. Vestiture of elytra (Fig. 3D) long (0.5–0.7 length of discal setae). Elytral chaetotaxy typical for *Anillinus*, umbilicate series of type A (*sensu*Jeannel 1963a and Giachino and Vailati 2011).

**Figure 4.**
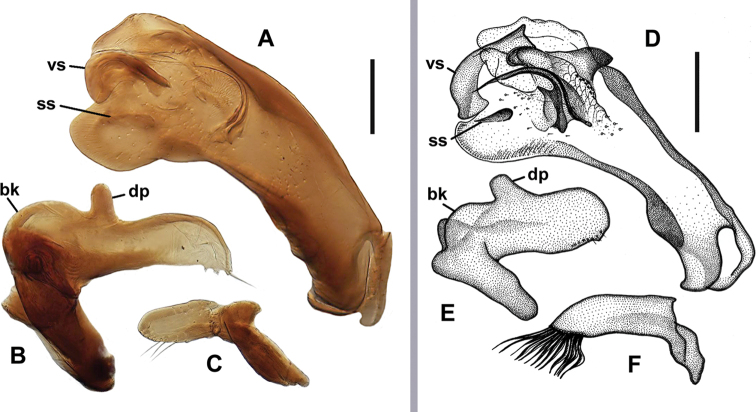
Digital images and line drawings of male genitalia of *Anillinus* species. *A.relictus* (E of Oneonta, Blount County, Alabama) **A** median lobe, right lateral aspect **B** left paramere, left lateral aspect **C** right paramere, right lateral aspect. *A.sinuatus* (Bexar County, Texas) **D** median lobe, right lateral aspect **E** left paramere, left lateral aspect **F** right paramere, right lateral aspect. bk – basal keel, dp – dorsal process, ss – spine-like structure, vs – ventral sclerite. Scale bars: 0.1 mm.

**Figure 5.**
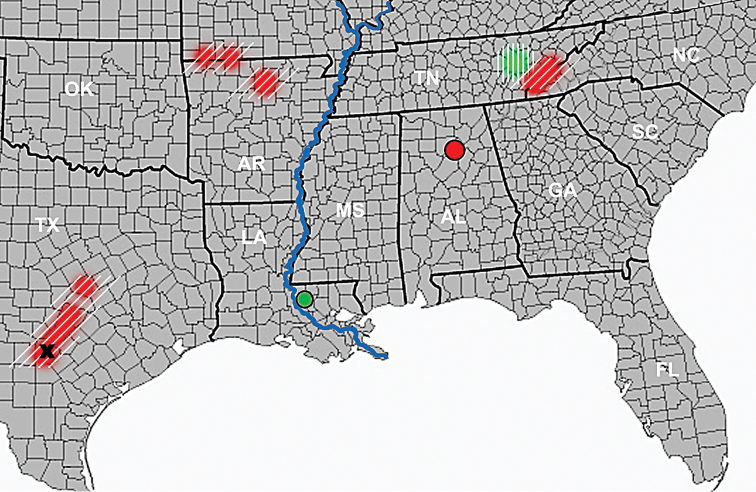
Map of the South of eastern United States, showing positions of locality records for the newly described species of *Anillinus* and the ranges of their presumed relatives (localities of the same color reflect supposed relatedness). *A.felicianus*, green circle; *A.relictus*, red circle. Green area with vertical line pattern – range of *A.sinuaticollis*. Black cross – type locality of *A.sinuatus*. Red areas with diagonal line pattern – ranges of the species of *Anillinus* whose males have a spine-like structure in the endophallus of the median lobe (after Sokolov et al. 2004; Sokolov and Watrous 2008; Sokolov 2011; Sokolov et al. 2014; Sokolov et al. 2017). Blue line – Mississippi River. State abbreviations follow Federal Information Processing Standards (https://www.nlsinfo.org/content/cohorts/nlsy79/other-documentation/codebook-supplement/nlsy79-attachment-102-federal).

***Prothorax***: Pronotum (Fig. 3C) moderately convex, of moderate size (LP/LE 0.40±0.003) and moderately transverse (WPm/LP 1.26±0.028), with lateral margins almost rectilinearly and slightly constricted posteriorly (WPm/WPp 1.19±0.013). Anterior angles slightly prominent, posterior angles nearly rectangular (95–100°). Width between posterior angles much greater than between anterior angles (WPa/WPp 0.87±0.038). Basal margin slightly concave in middle.

***Scutellum***: Externally visible, triangular, with rounded apex.

***Elytra***: Narrowly depressed along suture, of average length (LE/ABL 0.59±0.005) and width (WE/LE 0.66±0.026) for genus, with traces of 6–7 striae. Humeri distinct, rounded, in outline forming obtuse angle with longitudinal axis of body. Lateral margins subparallel in middle, slightly convergent at basal fifth, evenly rounded to apex at apical fourth, with shallow subapical sinuation. Basal margination distinct.

***Legs***: Protarsi of male with moderately dilated tarsomere 1. Profemora moderately swollen. Metafemora unmodified.

***Male genitalia***: Median lobe of aedeagus (Fig. 4A) anopic, slightly arcuate and slightly twisted. Basal orifice comparatively short for the genus. Shaft with long subparallel basal part, slightly dilating in apical third. Apical part with enlarged apex in form of rounded parallelogram. Dorsal margin slightly convex and strongly sclerotized at middle. Ventral margin curved near middle, where it is suddenly enlarged right before the apex. Endophallus with dorsal sclerite in form of a semicircular filament-like structure with short basal prolongations. Ventral sclerite located at apical orifice, in form of golf gap wedge plate. Dorsal scaly membranous field present at middle of dorsal sclerite. Enlarged apical area of median lobe with a dark spine-like structure (Fig. 4A, ss). Left paramere (Fig. 4B) modified, with long, subparallel apical half of moderate width with one seta at angulate tip, basally with strong concave keel (bk), and thick basal processes of different length. Right paramere (Fig. 4C) of moderate length, with eight setiferous pores bearing only three long setae (several others might be broken), which are shorter than length of paramere.

***Female genitalia***: Spermatheca not investigated. Ovipositor sclerites standard for genus with falciform gonocoxite 2 bearing two ensiform setae. Laterotergite with 8–9 setae.

###### Geographic distribution.

This species is known only from the type locality in Blount County, Alabama (Fig. 5, red circle).

###### Habitat.

The label does not contain any habitat information. Presumably, this species is not a cavernicolous species.

###### Relationships.

Based on the structure of the median lobe, *A.relictus* is a sister species to the endogean *A.sinuatus* (Jeannel) (Jeannel 1963a). The latter species is known to occur in Bexar County, Texas, where it was documented by a small series of three specimens extracted from the soil during surveys in peach orchards (Jeannel 1963a; Sokolov et al. 2014). The range of *A.sinuatus* (Fig. 5, black cross) is situated approximately 770 miles southwest of the type locality of *A.relictus*.

### The following key to the Alabama *Anillinus* is modified from couplet 2 in Sokolov (2020) to accommodate *A.relictus*:

**Table:** 

2(1)	Ventral parts of body (meso- and metathorax, abdominal sterna) covered with numerous setae. Spines of endophallus clustered together in a robust plate (Fig. 5G in Sokolov 2012, p. 67)	***A.hirsutus* Sokolov**
2'	Ventral parts of body without unusual vestiture. Spines of endophallus either lacking or, if present, scattered inside median lobe and separate from each other	**3a**
3a (2')	Elytra covered with shorter vestiture. Discal setae approximately 2.5–3.0 times longer than surrounding vestiture. Endophallus of median lobe lacking sclerotized spine-like structure in apical area and with copulatory sclerites merged into one structure	**3**
3a'	Elytra covered with longer vestiture. Discal setae at most 2 times longer than surrounding vestiture. Endophallus of median lobe with sclerotized spine-like structure in apical area and with two distant copulatory sclerites	***A.relictus* Sokolov, sp. nov.**
3(2')	[continue following key in Sokolov (2020)].	

## Discussion

The discovery of two new species extends our knowledge of the *Anillinus* fauna, the relationships within the genus, the distribution patterns of its representatives, and the evolutionary history of species inhabiting the Gulf Coast of the United States, a territory with a much less known fauna of *Anillinus* in comparison with the same fauna of the southern Appalachians.

Especially amazing is the discovery of *A.relictus*, because the immediate relatives of this Alabama species live in Texas. For *A.relictus*, some peculiar characters of the endophallus of the median lobe can be traced in several groups of *Anillinus* species separated geographically. At least three groups of species can be distinguished: (1) all Texan species except *A.depressus* Jeannel and *A.acutipennis* Sokolov and Reddel (whose males are still unknown), (2) both species from the Ozark Mountains, and (3) all three species of the *moseleyae*-group from the high altitudes of the Great Smoky Mountains. All these species have a unique character: the presence of a sclerotized, spine-like structure visible in the apical area of the median lobe (Fig. 4A, D ss). In addition, all species show modifications in the shape of the left paramere (Fig. 4B, E): a long right basal process and strong convex basal keel (bk), whose convexity shifts the position of the dorsal process (dp) of the paramere distally from its standard position. The eastern species of the *moseleyae*-group and *A.aleyae* Sokolov & Watrous from Missouri are closer to each other in having only one dorsal copulatory sclerite in the endophallus, an evenly-curved ventral margin, and the shorter shaft of the median lobe (Sokolov and Watrous 2008; Sokolov 2011). The Texan species and *A.alleni* Sokolov & Carlton, contrary to the members of the previous subgroup, have a longer shaft, often with a strong dorsal sclerotization, and more than one copulatory sclerite in the endophallus of the median lobe (Sokolov et al. 2014, 2017). Of these, *A.relictus* is morphologically closer to the Texan species and *A.alleni*, thus being the only representative of this subgroup east of the Mississippi River. Especially striking is the similarity in the structures of the median lobes between *A.relictus* and the endogean *A.sinuatus*. Similarities can be seen in the general proportion and the shape of the median lobe, in the similar dorsal sclerotization, and in the position and shape of the ventral sclerite (cf. Fig. 4A, D vs). Convergence in such a number of details within the male genitalia seems less probable than structural similarity due to the common origin of these two species. If this is correct, we can assume that at a certain point in time, their ancestor occupied a vast territory from the southern edges of the Cumberland Plateau westward to the Edwards Plateau. During glacial cycles, most migration routes in eastern North America followed the north-south direction, because of the local topography, especially that of the Appalachian Mountains, characterized by a series of parallel north-east to south-west trending valleys and ridges (Howden 1969; Gonzales et al. 2008). The directionality of glacial drainages was similar, and together, this topography slowed down the latitudinal migrations, brought genetic discontinuity, and formed east-west phylogeographical patterns in the southeastern USA (Liu et al. 2006; Pauly et al. 2007; Gonzales et al. 2008). This presumably prevented the formation of latitudinal ranges. Hence, it is reasonable to suppose that such a latitudinal range of the wingless ancestor of *A.sinuatus* and *A.relictus* likely arose before the Quaternary glaciations. Thus, the assemblage of species with a sclerotized, spine-like structure in the endophallus of the median lobe represents one of the ancient lineages of *Anillinus*.

The discovery of a new species of *Anillinus* in Louisiana is of biogeographical significance, because Louisiana is the only Gulf state in which anillines had previously never been recorded; thus, this record fills a gap in the distribution of *Anillinus* in the territories around the Gulf of Mexico. Speculating about the possible ways *A.felicianus* could have arisen in the territory of Louisiana, it is worth paying attention to the history of the Tunica Hills, the area where the species was discovered. Interestingly, many northern disjuncts of plants have been recorded to occur in this area (Delcourt and Delcourt 1996). It was once considered as an isolated “island” refugium for plant species, which presumably had migrated southward during the glaciation cycles (Braun 1950). The majority of these plants now occur within north-central United States and in the Appalachian Mountains (Delcourt and Delcourt 1975). Thus, during the glacial cycles, some of the Appalachian plant communities probably reached the Tunica Hills area. Evidently, other living organisms associated with plant communities could have migrated synchronously. The recent discoveries of the eastern Bembidion (Hirmoplataphus) nigrum Say (Bousquet 2012) and *Batrisodesdorothae* Ferro & Carlton (Ferro and Carlton 2014) in the Tunica Hills may constitute a strong evidence in support of this assumption. Therefore, the external similarity between *A.felicianus* and the Appalachian endogean *A.sinuaticollis* was, in fact, not unexpected. Unfortunately, the males of *A.sinuaticollis* are still unknown, making it impossible to examine male genitalia of this species. However, many of the characters of the male genitalia of *A.felicianus*, including its blade-like dorsal sclerite with a basally attached ventral plate of the endophallus of the median lobe, can be found in the median lobes of another group of Appalachian *Anillinus* – the litter members of the *langdoni*-group (Sokolov et al. 2007). Both of these facts unequivocally point to the Appalachian origin of *A.felicianus*. It is likely that, contrary to *A.relictus*, the speciation of *A.felicianus* happened comparatively late, presumably during the Quaternary glacial cycles, although the details remain obscure. Given its external morphology, the closest relative of the endogean *A.felicianus* should be the endogean *A.sinuaticollis*, not the litter species of the *langdoni*-group. In contrast to the litter representatives of the *langdoni*-group, both endogean species lack the microsculpture on the pronotal disc and have slightly different body proportions. Whether these characters are adaptive and repeatedly evolve each time the litter species developed adaptations to living in a subterranean environment is not clear. Currently, both scenarios of the arising of *A.felicianus* should be considered as equally possible: (1) an allopatric origin after splitting the range of migrated endogean species, or (2) a sympatric origin from migrated litter species, whose adaptations to an endogean lifestyle gave birth to a new species, while the ancestral litter form subsequently became extinct due to unknown, possibly ecological factors.

## Supplementary Material

XML Treatment for Anillinus

XML Treatment for Anillinus felicianus

XML Treatment for Anillinus relictus
